# Progesterone ameliorates diabetic nephropathy in streptozotocin-induced diabetic Rats

**DOI:** 10.1186/s13098-015-0097-1

**Published:** 2015-11-14

**Authors:** Bahaa Al-Trad, Ibraheem M. Ashankyty, Mohd Alaraj

**Affiliations:** Department of Biological Sciences, Yarmouk University, Irbid, Jordan; Department of Physiology, College of Medicine, University of Ha’il, 2440 Hail, Saudi Arabia; Department of Clinical Laboratory Sciences, College of Applied Medical Sciences, University of Ha’il, Ha’il, Saudi Arabia; Department of Medical Laboratory Technology, Faculty of Applied Medical Sciences, King Abdulaziz University, Jeddah, Saudi Arabia; Department of Pharmacology, College of Medicine, University of Ha’il, Hail, Saudi Arabia

**Keywords:** Progesterone, Diabetic nephropathy, Fibrosis, Angiogenesis, Angiotensin II type 1 receptor

## Abstract

**Background:**

Previous studies reported that 17β-estradiol may influence the progression of diabetic renal disease in females. The present study was intended to provide an insight into the specific effects of progesterone, the other female sex hormone, in the diabetic renal complications.

**Methods:**

Adult female wistar rats were divided into four groups (n = 6/group): intact control (non-diabetic, ND), intact diabetic (D), ovariectomized diabetic (D-OVX) and ovariectomized diabetic which were treated with progesterone (D-OVX + P; 10 mg/kg, s.c, every second day) for 10 weeks. Diabetes was induced by a single dose injection of 55 mg/kg streptozotocin. Expressions of transforming growth factor-β (TGF-β), fibronectin, vascular endothelial growth factor-A (VEGF-A), angiotensin II type 1 receptor (ATR1) and podocyte markers (nephrin and podocin) were assessed by immunohistochemistry and real-time PCR.

**Results:**

The treatment of D-OVX rats with progesterone attenuated diabetic-associated increases in the urinary albumin to creatinine ratio, glomerulosclerosi and the expression of profibrotic and angiogenic factors (TGF-β, Fibronectin and VEGF-A). Furthermore, progesterone supplementation prevented diabetes-induced downregulation of nephrin and podocin while the overexpression of ATR1 in the diabetic rats was inhibited by the progesterone supplementation.

**Conclusion:**

These results provided evidence, for the first time, that the replacement of progesterone can ameliorate the renal damage in the experimental models of diabetic nephropathy through improving the renal function; the inhibition of renal fibrosis and abnormal angiogenesis; along with the amelioration of podocyte injury. Additionally, the blocking of renin-angiotensin system through the down-regulation of ATR1 expression may also account for the reno-protective effect of progesterone.

**Electronic supplementary material:**

The online version of this article (doi:10.1186/s13098-015-0097-1) contains supplementary material, which is available to authorized users.

## Background

Among the diabetic complications, the diabetic nephropathy (DN) is the single most common cause of end-stage renal failure worldwide, and a major indication for dialysis and transplantation [1, 2]. DN is mainly caused by haemodynamic and structural changes including glomerular hyperfiltration and hypertrophy, thickening of glomerular basement membrane, expansion of mesangial extracellular matrix and fibrosis that is associated with decline of glomerular filtration rate and substantial proteinuria [2]. In spite of the number of studies on human and rodent models of experimental DN, effective therapy is not available yet. Consequently, the search for drugs to blockade of the progression of DN has high priority in biomedical research.

Increasing evidence suggests that non-diabetic premenopausal women have a lower incidence, prevalence, and progression of many renal diseases compared to age-matched non-diabetic men [3, 4]. These observations suggest that female gender may be protective, and that male gender may be a risk factor for the development and progression of non-diabetic renal disease [3, 5]. However, insight from in vitro studies and animal models suggest that diabetes is a state of an imbalance in sex steroids hormone levels [5]. Experimental and clinical studies showed that this imbalance in sex hormone levels, decreased estradiol and increased testosterone, in female streptozotocin (STZ)-induced diabetic rats is associated with the progression of DN, which support the notion that sex hormones may play a role in the pathogenesis of diabetic renal disease [5, 6]. Reduced levels of plasma estradiol and imbalance in the renal expression of estrogen receptors may explain why female gender is no longer a renoprotection factor in the setting of diabetes mellitus [7]. This hypothesis is in line with previous studies reporting that restoring estradiol levels in STZ-induced diabetic rats by 17β-estradiol replacement is renoprotective by attenuating the decline in renal function and pathology associated with DN [6, 8].

What remains unclear is the role that progesterone, the other female sex hormone, plays in the pathology of the DN. In thekidney, progesterone receptors have been found mainly in distal tubule cells, although they are present in cortex and medulla of males and females kidneys at the level of transcription and translation [5, 9]. In the ovariectomized rats with renal ablation, estradiol protected against proteinuria and glomerulsclerosis, but the rats treated with estradiol plus progesterone exhibited the same renal damage as vehicle-treated rats [10]. These findings suggest that the presence of progesterone may attenuate the beneficial effects of estradiol in that experiment. However, in other studies, renal injuries of various etiologies were ameliorated by progesterone. The age-dependent decrease in the renal functional reserve and proximal tubular fluid output in the ovariectomized rats were prevented by estradiol alone or estradiol and progesterone [11]. Similarly, in ischemia–reperfusion–induced acute kidney injury, the exogenous administration of progesterone exerts significant antioxidant and renoprotective effects in a dose-dependent manner [12]. Finally, in the DOCA salt hypertension rats, the treatment with estrogen plus progesterone or progesterone, but not estrogen alone, attenuated renal damage [13].

Similar to estrogen, diabetic females also exhibit a decrease in progesterone levels [14]. However, no studies to date have addressed the effects of progesterone in DN. Therefore, the present study was intended to provide insight into the specific effects of progesterone in diabetic renal complications. We demonstrate that in the STZ-induced diabetic rat, replacement with progesterone for 10 weeks is renoprotective, by improvement of renal functional and structural changes and inhibition of fibrosis.

## Methods

### Induction of diabetes and experimental protocols

All experimental procedures were pre-approved by the university animal care and use committee (Protocol number: 16/3/3/257). The study was performed in female adult Wister rats, 55–60 days old and weighing approximately 200 g. The rats were housed in a controlled environment at 21–23 °C on an illumination schedule of 12 h of light and 12 h of darkness. Standard pellet food and water were provided ad libitum. All groups were feed the recommended diet for maintenance with 5 % total fat and 18 % total protein.

Diabetes was induced in rats by intera-peritoneal injecting a freshly prepared STZ) Sigma-Aldrich, USA; 55 mg/kg; dissolved in 0.1 M acetate buffer; pH 4.5) after an overnight fast. A control group of 6 rats received citrate buffer only. The diabetic rats were ovariectomized two days after the STZ injection, once serum glucose is higher than 300 g/dl. The procedure for ovariectomy followed the method of Khajuria et al. [15]. The animals were anesthetized with a combination of ketamine/xylazine anesthesia (100/10 mg/kg, i.p.). After the anesthesia was confirmed, the area of surgery was cleaned with ethanol and a single ventral transverse incision (0.5 cm) was made through the skin and the bilateral ovaries and the ovarian fats were located. Then, ovaries were isolated by ligation of the most proximal portion of the oviduct before removal. The surgical incision was sutured and the animals were reversed to their cages to recover from surgery.

After that, the rats were randomly divided into four treatment groups (n = 6 per group): (1) intact control (non-diabetic, ND), (2) intact diabetic (D), (3) ovariectomized diabetic (D-OVX) and (4) ovariectomized diabetic which were treated with progesterone (D-OVX + P). Progesterone (10 mg/kg, Sigma-Aldrich, USA) was dissolved in sesame oil (Sigma-Aldrich, USA) and administered subcutaneously every second day for 10 weeks after induction of diabetes [10]. The ND, D and D-OVX groups were received the vehicle only. Rats were weighed every week and the dose of administration was adjusted according to recorded body weight.

### Urine, blood and tissue collection

At the end of 10 weeks of treatment, the rats were placed in metabolic cages 1 day before sacrifice, and urine was collected for 24 h for the analysis of urine albumin concentration and the urine output. Then, the animals were weighed and anesthetized with ketamine and xylazine (100/10 mg/kg, i.p.), and blood samples were collected (via cardiac puncture). The right kidney was removed and then transferred into RNAlater solution (Sigma-Aldrich, USA) for the real time PCR analysis. The left kidney was fixed with 4 % paraformaldehyde for morphological and immunohistochemical analysis. The animals were sacrificed via anesthetic overdose.

### Measurements of blood glucose, urinary albumin to creatinine ratio and serum progesterone levels

the blood glucose level was determined by glucometer (Accu-Chek Performa, Roche Diagnostics). Urine samples were centrifuged at 4 °C and 2000 rpm for 10 min. The urinary albumin and creatinine concentrations in the supernatant were measured using Albumin Rat ELISA kit (Abcam, UK) and creatinine ELISA kit (Cusabio, China) according to the manufacturer’s protocols. The urinary albumin to creatinine ratio (UACR) was calculated based on the albuminuria and urine creatinine level (albumin mg/creatinine g) [16]. Serum progesterone and estradiol levels were measured by commercially available ELISA kits (Biocheck, Inc. Foster City CA, USA and Cusabio, wuhan, China, respectively), according to the manufacturer’s protocols.

### Estimation of glomerulosclerosis

After the fixation in 4 % paraformaldehyde, the tissues were processed to paraffin, sectioned at 4 μm, and stained with periodic acid Schiff (PAS) using PAS stain Kit (Abcam, UK) according to the manufacturer’s protocols. One hundred glomeruli per section were randomly selected and the PAS-stained sections were examined by observer masked to the treatment groups using the light microscope. The degree of glomerular damage was assessed using a semi-quantitative scoring method: grade 0, normal glomeruli; grade 1, sclerotic area up to 25 % (minimal sclerosis); grade 2, sclerotic area 25–50 % (moderate sclerosis); grade 3, sclerotic area 50–75 % (moderate-severe sclerosis); grade 4, sclerotic area 75–100 % (severe sclerosis). The glomerulosclerotic index (**GSI**) was calculated using the following formula: GSI = (1 × n1) + (2 × n2) + (3 × n3) + (4 × n4)/n0 + n1 + n2 + n3 + n4, where nx is the number of glomeruli in each grade of glomerulosclerosis [17].

### RNA preparation and reverse transcription

Total RNA was extracted from RNAlater-preserved kidney tissues using an RNeasy mini tissue kit (Qiagen, USA) according to the manufacture protocols. The resulting RNA pellets were dissolved in RNase-free water and the quantity and quality of the isolated RNA were determined by absorbance at 260 and 280 nm and OD 260/280 nm ratios >1.8 were obtained for all samples, indicating high purity. Samples were then stored at −20 °C for subsequent RT-PCR analysis.

Total RNA (0.5 µg) was reversely transcribed using oligo-(dT)15 primer in a 20-µl reaction according to the manufacturer’s instructions (iNtRON Biotechnology, S.Korea). Reverse transcription reactions were carried out at 25 °C for 10 min followed by 42 °C for 60 min and 95 °C for 5 min. The resulting first-strands cDNA were stored at −20 °C until use for real time RT-PCR.

### Real time RT-PCR

Quantitative real time RT-PCR was carried out on LineGene 9600 Real-Time PCR system (Bioer Technology Co, Bingjiang, China), using β-actin as a non-regulated reference gene. Primers were designed and synthesized by IDT (Integrated DNA Technologies, INC., IA; Additional file 1: Table S1). The SYBR-PCR reactions were performed using the KAPA SYBR^®^ FAST Universal 2X qPCR master mix (KAPA Biosystem, USA) in 10 μl final volume. The amplification conditions for quantification were: 95 °C for 3 min and 45 cycles of 95 °C for 3 s and 60 °C for 20 s. After the amplification efficiency of each target and reference gene was validated, the relative gene expression levels were determined by the ^ΔΔ^CT method as described by Livak and Schmittgen [18]. The levels of genes expression were expressed as the normalized ratio of gene expression relative to β-actin mRNA level using one sample from the control group as calibrator.

### Immunohistochemistry

Immunohistochemical staining was performed with the ImmunoCruz Rabbit/Mouse ABC Staining System Kit (Santa Cruz, USA) according to the manufacture protocol. In brief, mounted sections were deparaffinized in xylene and rehydrated in descending series of alcohol concentrations. In order to expose the masked antigenic sites, sections were subjected to autoclave at 121 °C in citrate buffer (10 mM sodium citrate pH 6.0) for 10 min and allowed to cool at room temperature for 20 min. To quench endogenous peroxidase activity, sections were incubated in 1 % methanolic H_2_O_2_ for 20 min. Non-specific binding sites were blocked with protein block for 30 min. Sections were then incubated with specific primary antibodies against: TGF-β (1:100; Santa Cruz/USA; Cat.No.sc-146), Fibronectin (1:250; Abcam/UK; Cat.No.ab2413), matrix metalloproteinase-2 (MMP-2; 1:100; Santa Cruz/USA; Cat.No. sc-13595), angiotensin II (Ang II) type I receptor (ATR1; 1:100; Santa Cruz/USA; Cat.No.sc-1173), vascular endothelial growth factor-A (VEGF-A; 1:100; Santa Cruz/USA; Cat.No.sc-7269) and podocyte markers (nephrin and podocin) proteins (1:100; Abcam/UK; Cat.No.ab183099 and Cat.No.ab50339, respectively). For negative controls, the primary antibody was omitted. At the end of each primary antibody incubation period, the sections were washed three times with phosphate buffer saline (PBS) and sections were then incubated with biotinylated secondary antibody for 30 min at room temperature in a humidified chamber. The sections were then washed three times with PBS and incubated with AB enzyme reagent for 30 min at room temperature in a humidified chamber. The immunoreactions were developed by incubate the sections in 1–3 drops peroxidase substrate. The reaction was terminated in distilled water, and the sections were counterstained with Mayers haematoxylin, dehydrated and mounted.

### Statistical analysis

All data will be expressed as mean ± SEM. One-way analysis of variance (ANOVA) was used to identify differences between groups. When this indicated significance (*P* < 0.05), Tukey post hoc test analysis was used to determine which conditions are significantly different from each other. All statistical analysis was performed using SPSS version 14.0 for Windows (SPSS Inc., Chicago, IL, USA).

## Results

### Metabolic and physiological parameters in control and diabetic rats

As shown in Table 1, after 10 weeks of diabetes blood glucose was significantly higher in the D than in the ND rats. A similar increase in blood glucose was observed in the diabetic group with OVX, and OVX + P supplementation. The diabetic rats showed a significant decrease in body weight (Additional file 2: Fig. S1; *P* < 0.05) as compared with the ND group. However, no differences in body weight were observed between the intact, the D-OVX, or the D-OVX + P in the diabetic groups. A significantly increased level of UACR (Table 1; *P* < 0.05) was evident in the D group compared to the ND group, with the exacerbation in the D-OVX rats. The treatment of the diabetic rats with progesterone significantly recovered the UACR (Table 1; *P* < 0.05) in comparison with the D and the D-OVX groups, suggesting that the progesterone replacement attenuated the progression of the DN.

**Table 1:** Effects of progesterone supplementation on metabolic and renal parameters

	ND	D	D-OVX	D-OVX + P
Blood glucose, mg/dl	96.6 ± 4.8	454 ± 50.1*	476 ± 10.5*	481 ± 9.1*
Serum progesterone, ng/ml	29.9 ± 2.2	25.9 ± 0.6	13.2 ± 3.3*	32.1 ± 3.9^£^
serum estrogen, pg/ml	46.3 ± 2.9	40.2 ± 0.6	19.4 ± 0.09*	29.6 ± 3.4*
UACR, mg/g	20.5 ± 1.6	94.6 ± 12.9*	127.2 ± 25.8*	59.4 ± 12.9*^,#^

Data represent the mean ± SEM. * P < 0.05 compared to the ND group. ^#^P < 0.05 compared to D and OVX-D groups. ^£^ P < 0.10 compared to D group

*UACR* urinary albumin to creatinine ratio

There was a trend toward lower serum progesterone and estrogen levels in the D group compared to the ND rats, but this difference was not statistically significant (Table 1; *P* > 0.10). However, there was a tendency for a higher serum progesterone level in the D-OVX + P compared with the D group (Table 1; *P* < 0.10).

### Effect of progesterone replacement on glomerular hypertrophy and early markers of fibrosis

**Fig. 1:**
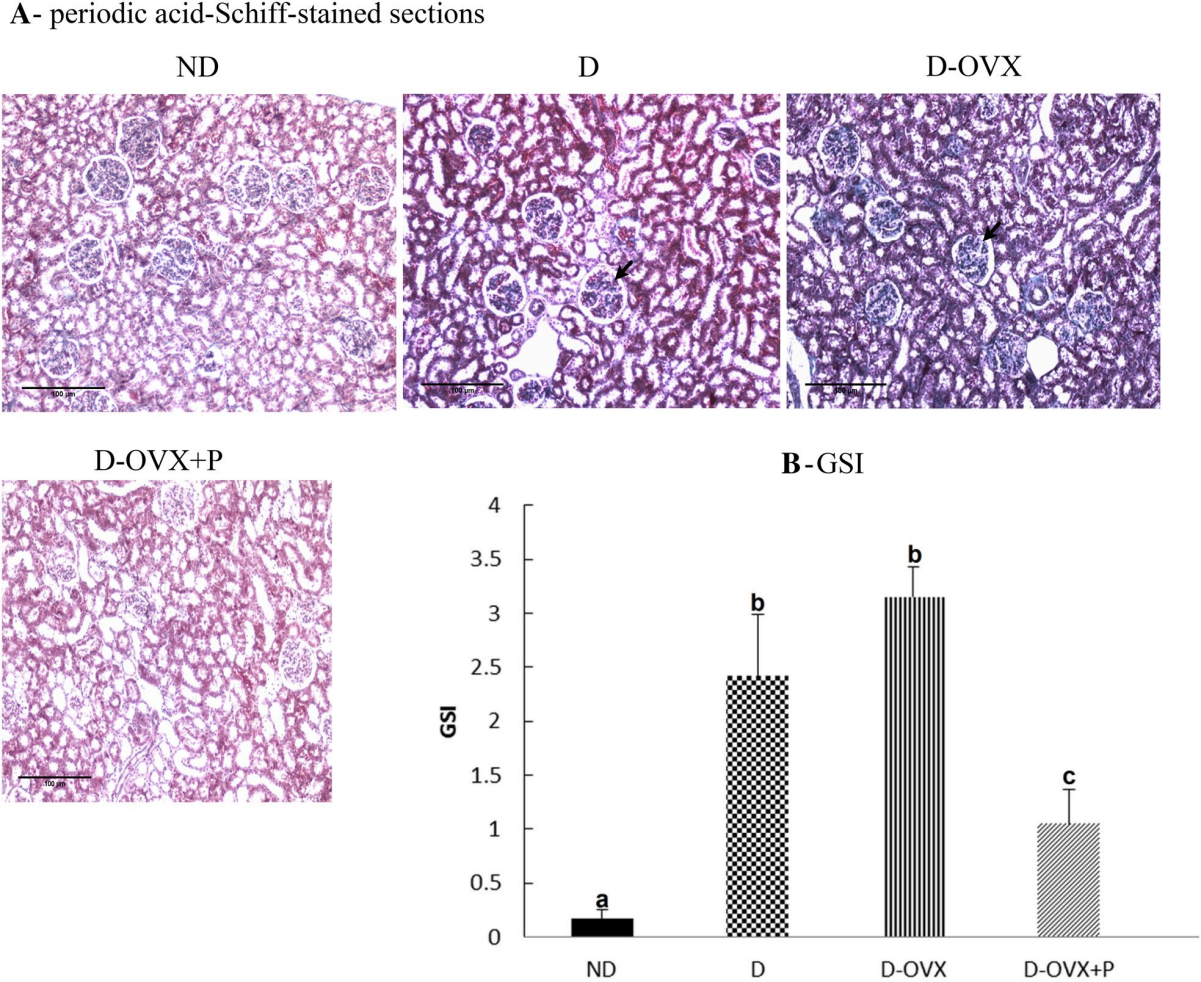
Periodic acid-Schiff (PAS) stained sections detect that progesterone replacement attenuated the glomerulosclerotic index (GSI) in the diabetic kidney. **A** PAS-stained sections (magnification, ×200) show that the percentage of the PAS-positive material, indicative of increased fibrosis, was significantly increased in the D and the D-OVX groups compared with the ND group (*arrows*). Progesterone replacement inhibited the increase in the PAS-positive material in the D + OVX + P group (**B**) GSI. Data represent the mean ± SEM. Means with *different superscript letters* are significantly different from one another (*P* < 0.05)

At the end of 10 weeks of treatment, the diabetic rats with vehicle treatment showed a marked glomerular hypertrophy, a mesangial expansion and a clearly increased accumulation of extracellular matrix in the mesangium compared to ND. Consequently, the GSI of the D rats was significantly higher than ND controls (ND, 0.16 ± 0.08; D, 2.41 ± 0.57; *P* < 0.05; Fig. 1B). The D-OVX rats showed the highest GSI score while treatment of the D-OVX rats with progesterone ameliorated the above pathogenic findings.

**Fig. 2:**
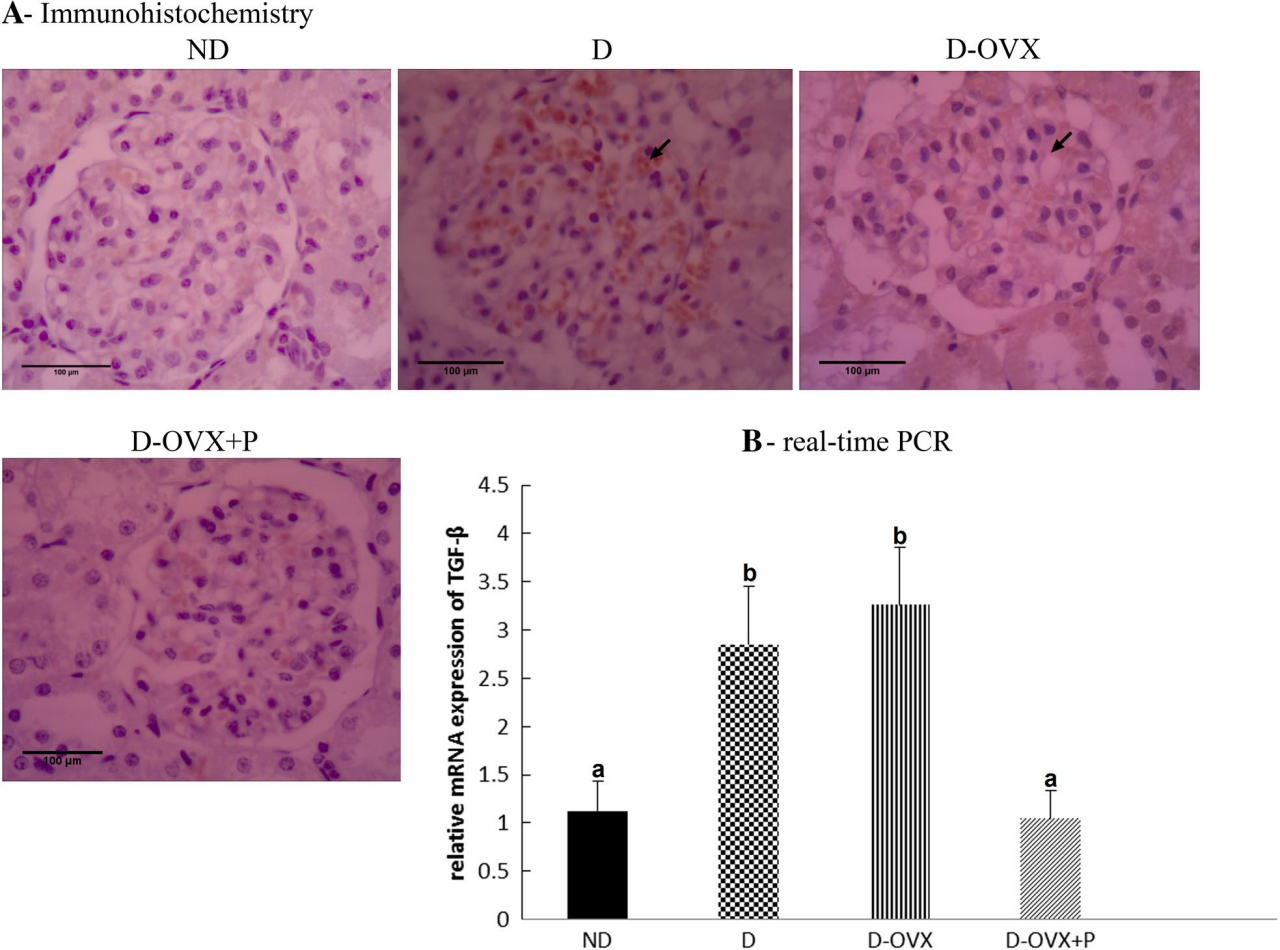
Immunohistochemistry and real-time PCR detect that progesterone replacement inhibits the TGF-β expression in the diabetic kidney. **A** immunohistochemical stain of the kidney sections (hematoxylin staining; magnification, ×400) show that the TGF-β immunostaining (brown staining) in the glomeruli was much stronger in the D and the D-OVX groups compared with the ND group (*arrows*). Progesterone replacement inhibited the increase in the TGF-β immunostaining in the D + OVX + P group. **B** TGF-β mRNA expression by real-time PCR. Data represent the mean ± SEM. Means with *different superscript letters* are significantly different from one another (*P* < 0.05)

**Fig. 3:**
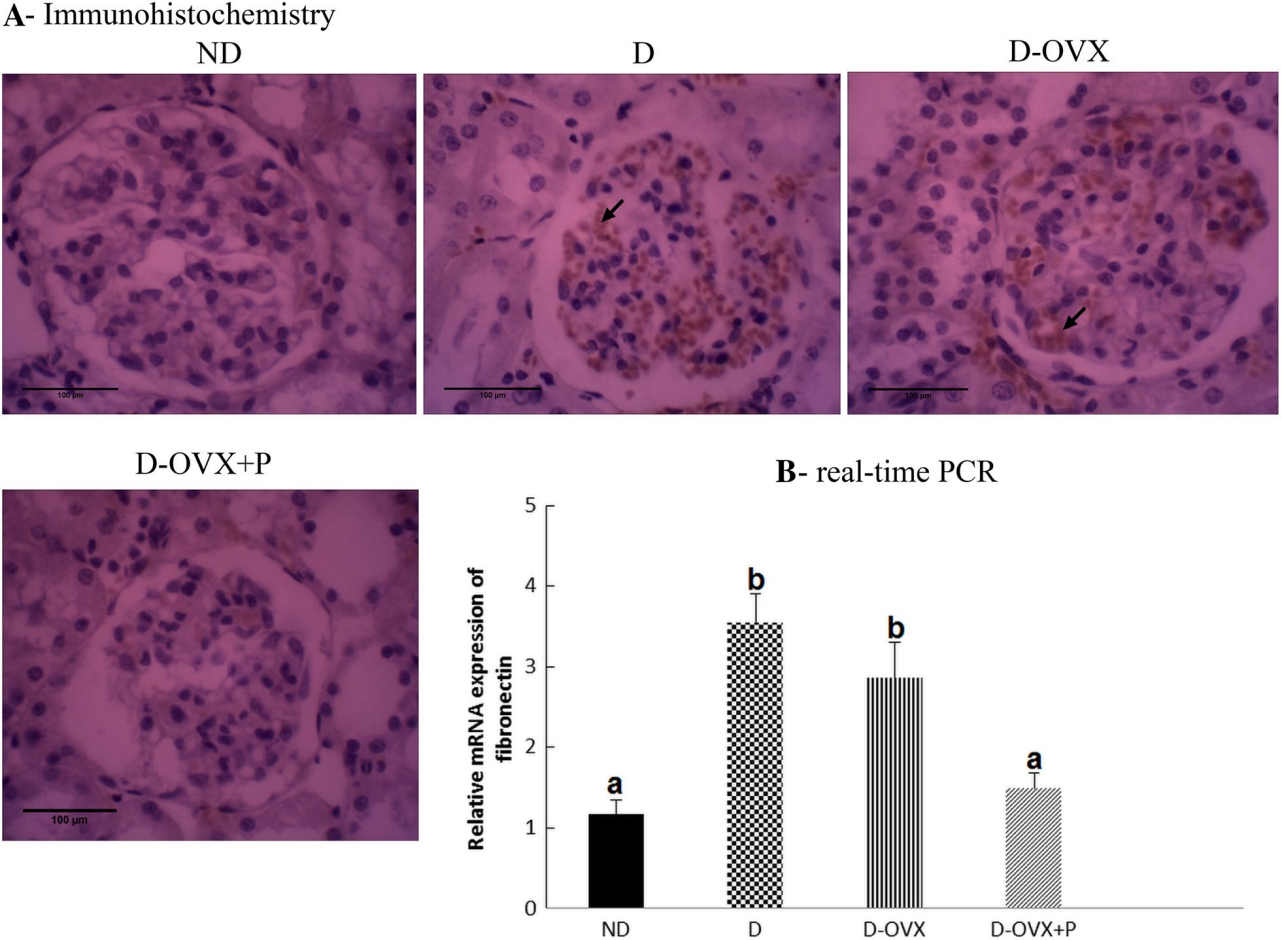
Immunohistochemistry and real-time PCR detect that progesterone replacement inhibits the fibronectin expression in the diabetic kidney. **A** immunohistochemical stain of the kidney sections (hematoxylin staining; magnification, ×400) show that the fibronectin immunostaining (brown staining) in the glomeruli was much stronger in the D and the D-OVX groups compared with the ND group (*arrows*). Progesterone replacement inhibited the increase in the fibronectin immunostaining in the D + OVX + P group. **B** fibronectin mRNA expression by real-time PCR. Data represent the mean ± SEM. Means with *different superscript letters* are significantly different from one another (*P* < 0.05)

To determine the effect of progesterone on extracellular matrix (**ECM**) proteins deposition in glomeruli, we analyzed the TGF-β and fibronectin expression in the in renal glomeruli in the diabetic kidney. As presented in Figs. 2A and 3A, the immunohistochemistry analysis demonstrated that diabetes was associated with an overall increase in the intensity of immunostaining for TGF-β and fibronectin in the D and D-OVX groups. Consistent with the immunohistochemical findings, the levels of mRNA encoding for TGF-β and fibronectin were significantly greater in the D and the D-OVX groups than in the ND group (Figs. 2B, 3B; *P* < 0.05). The treatment of D-OVX rats with progesterone decreased the over expression of these markers of fibrosis, suggesting that progesterone treatment inhibits the progression of renal fibrosis diabetic kidney.

**Fig. 4:**
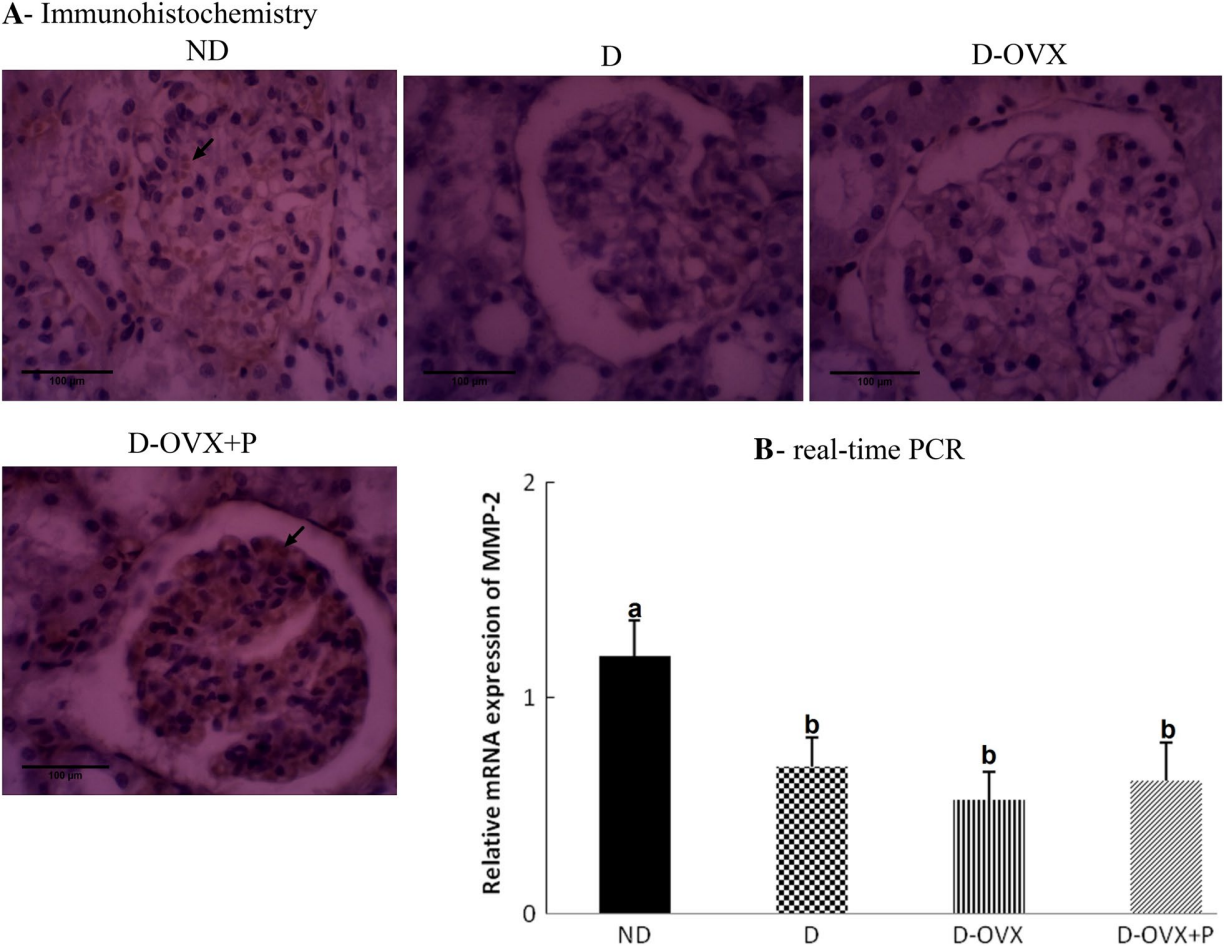
Immunohistochemistry and real-time PCR detect that progesterone replacement increase the MMP-2 protein, but not the mRNA levels in the diabetic kidney. **A** Immunohistochemical stain of the kidney sections (hematoxylin staining; magnification, ×400) show that the MMP-2 immunostaining (brown staining) in the glomeruli was much stronger in the ND group compared with the D and the D-OVX groups (*arrows*). Progesterone replacement inhibited the decrease in the MMP-2 immunostaining in the D + OVX + P group. **B** MMP-2 mRNA expressions by real-time PCR. Data represent the mean ± SEM. Means with *different superscript letters* are significantly different from one another (*P* < 0.05)

**Fig. 5:**
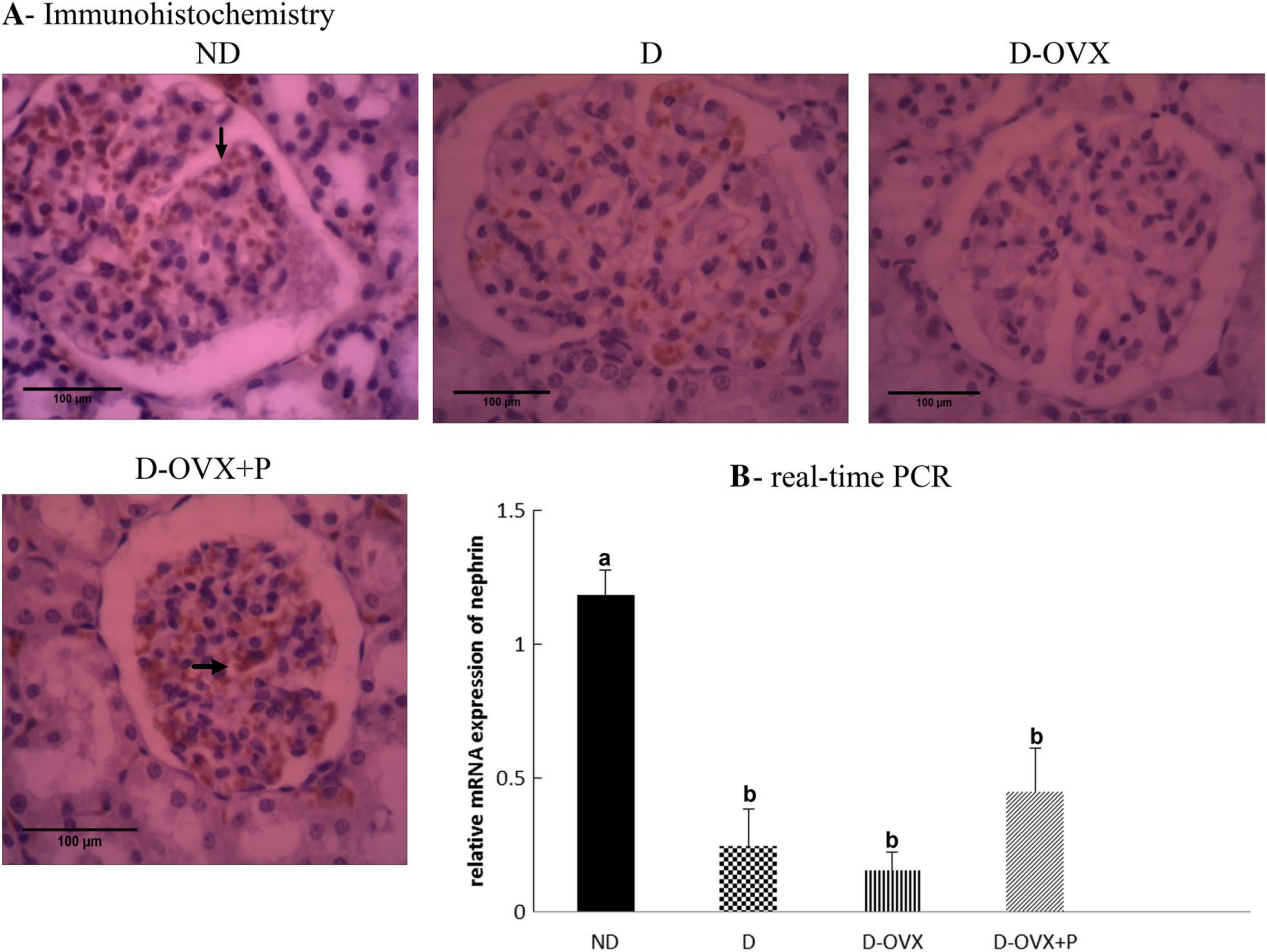
Immunohistochemistry and real-time PCR detect that progesterone replacement partially restores the nephrin expression in the diabetic kidney. **A** immunohistochemical stain of the kidney sections (hematoxylin staining; magnification, ×400) show that the nephrin immunostaining (brown staining) in the glomeruli was much stronger in the ND group compared with the D and the D-OVX groups (*arrows*). Progesterone replacement inhibited the decrease in the nephrin immunostaining in the D + OVX + P group. **B** nephrin mRNA expressions by real-time PCR. Data represent the mean ± SEM. Means with *different superscript letters* are significantly different from one another (*P* < 0.05)

It has been shown that the glucose-induced inhibition of matrix degrading enzymes such as MMP-2 is believed to contribute to accumulation of mesangial ECM proteins in the diabetic kidney [19]. Therefore, we determined whether the progesterone affects the expression of MMP-2 in the diabetic kidney or not. The results showed that the MMP-2 mRNA expression and the immunostaining intensity were decreased in the D and the D-OVX groups compared to the kidney of the ND group (Fig. 4A, B). The progesterone treatment did not affect significantly the MMP-2 mRNA levels in the diabetic kidney when compared to that in the ND control group (P > 0.05; Fig. 5B). However, the immunohistochemistry analysis demonstrated that the progesterone treatment increased the protein expression of MMP-2; suggesting a possible post-transcriptional regulation of the MMP-2 protein by the progesterone treatment.

### Progesterone restores the expression of protein and mRNA of podocyte markers (nephrin and podocin) in rats with the DN

**Fig. 6:**
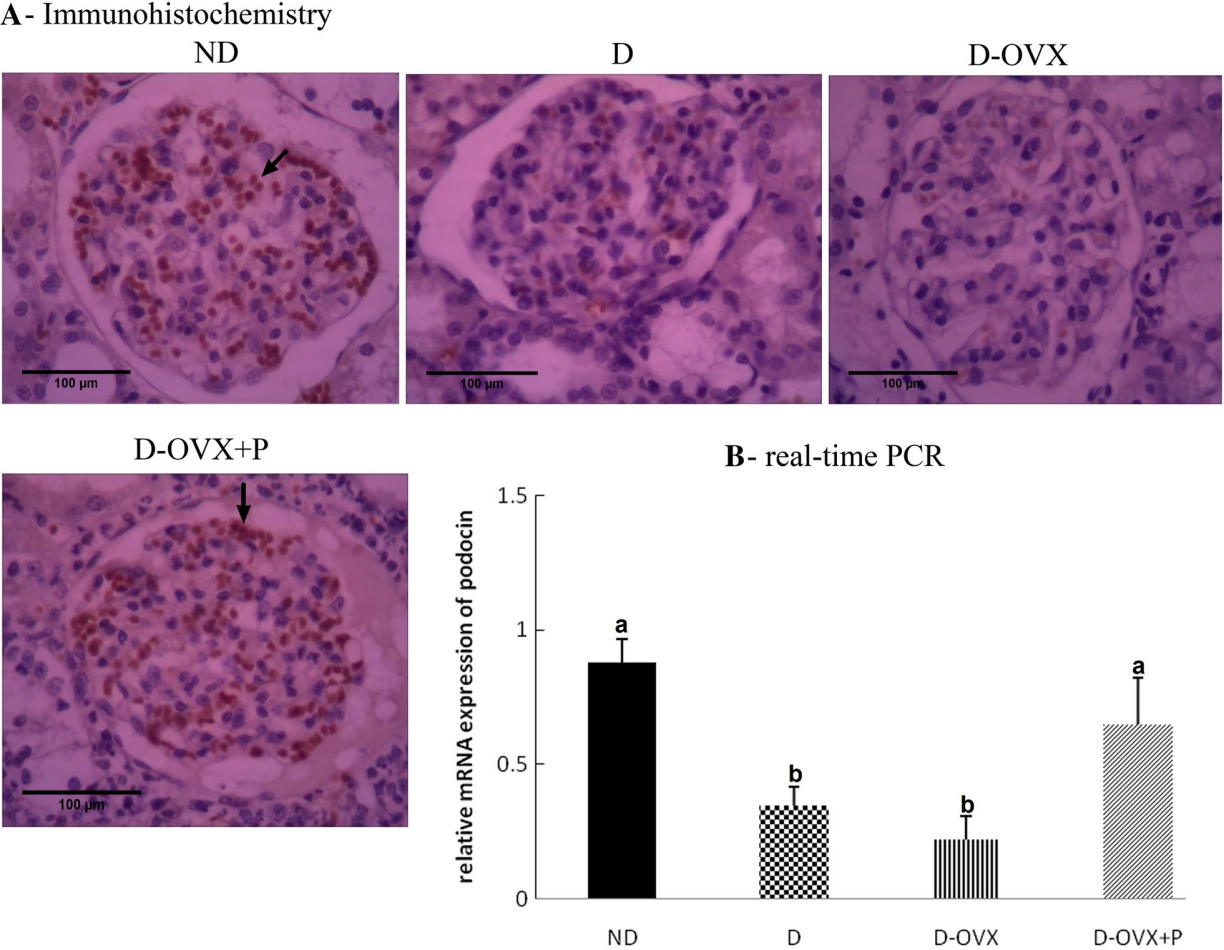
Immunohistochemistry and real-time PCR detect that progesterone replacement restores the podocin expression in the diabetic kidney. **A** Immunohistochemical stain of the kidney sections (hematoxylin staining; magnification, ×400) show that the podocin immunostaining (brown staining) in the glomeruli was much stronger in the ND group compared with the D and the D-OVX groups (*arrows*). Progesterone replacement inhibited the decrease in the podocin immunostaining in the D + OVX + P group. **B** Podocin mRNA expressions by real-time PCR. Data represent the mean ± SEM. Means with *different superscript letters* are significantly different from one another (*P* < 0.05)

The expression intensity and the distribution pattern of nephrin and podocin in glomeruli were observed by immunohistochemical staining (Figs. 5A, 6A). The staining of nephrin and podocin was prominent in the glomerular tufts in the ND rats. By contrast, in the D rats, the expression of nephrin and podocin was significantly decreased. This diabetes-associated decrease in the apparent intensity of the immunostaining of nephrin and podocin was exaggerated in the D-OVX group. Consistent with the expression intensity of nephrin and podocin, the expression levels of nephrin and podocin mRNA were significantly decreased in the kidney of the untreated D and D-OVX rats compared with the ND rats (Figs. 5B, 6B, *P* < 0.05). However, the progesterone replacement partially (nephrin) or fully (podocin) improved the immunohistochemical staining intensity and the mRNA expression of podocyte markers in the D-OVX + P group compared with the D and the D-OVX rats.

### Decreased ATR1 expression in the diabetic kidney by progesterone treatment

**Fig. 7:**
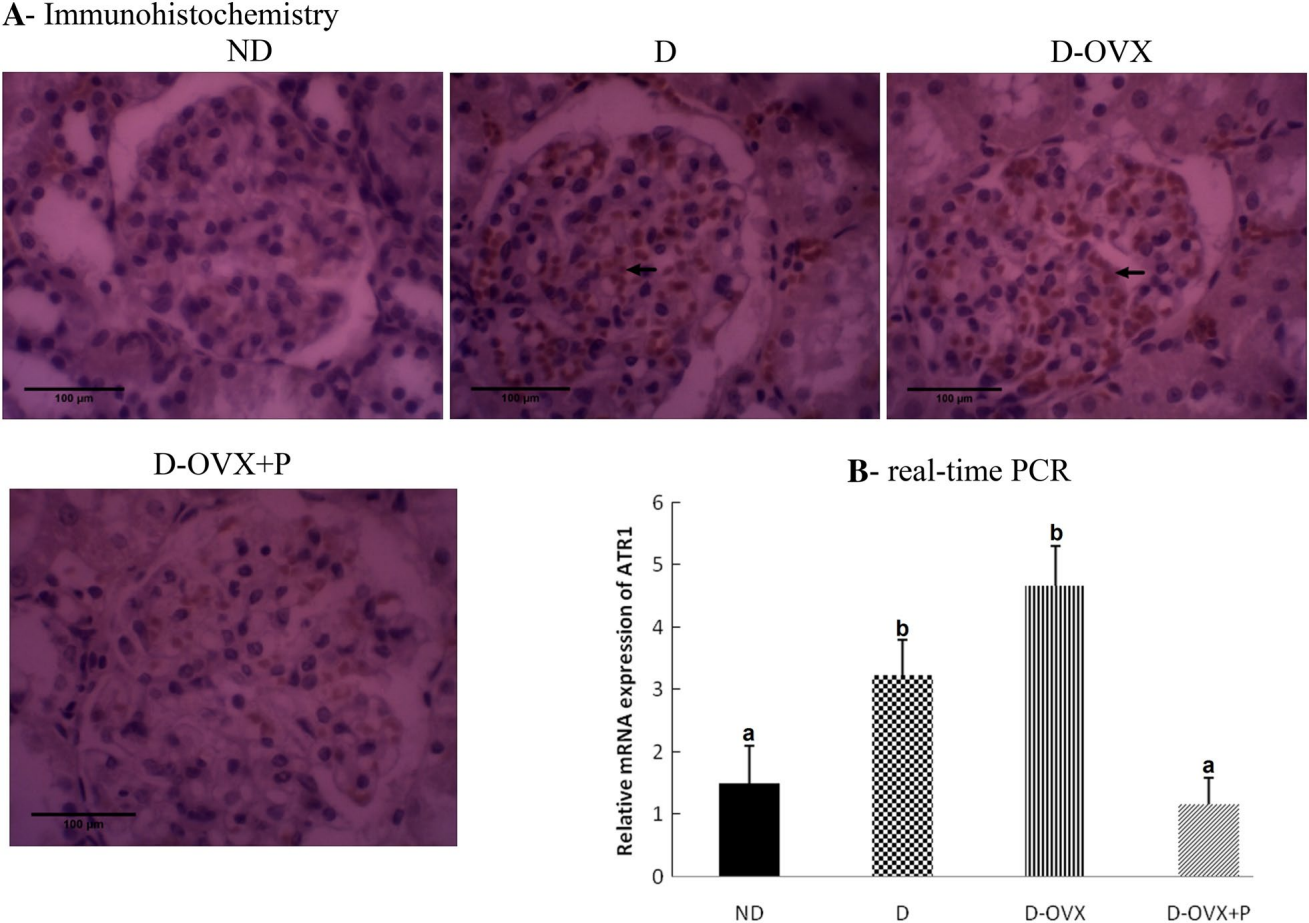
Immunohistochemistry and real-time PCR detect that progesterone replacement inhibits the AT1R expression in the diabetic kidney. **A** Immunohistochemical stain of the kidney sections (hematoxylin staining; magnification, ×400) show that the AT1R immunostaining (brown staining) in the glomeruli was much stronger in the D and the D-OVX groups compared with the ND group (*arrows*). Progesterone replacement inhibited the increase in the AT1R immunostaining in the D + OVX + P group. **B** AT1R mRNA expression by real-time PCR. Data represent the mean ± SEM. Means with *different superscript letters* are significantly different from one another (*P* < 0.05)

ATR1 overexpression plays an important role in the development and progression of the DN [20, 21]. Thus, we examined whether progesterone may protect against the renal injury via its effects on ATR1 protein and mRNA expression. The real-time PCR and immunohistochemistry showed that the ATR1 mRNA and the protein expression levels were high in both the D and the D-OVX rats comapred with the ND rats, and these changes were significantly attenuated by the progesterone replacment (Fig. 7A, B, *P* < 0.05).

### Progesterone modulated angiogenesis in the DN

**Fig. 8:**
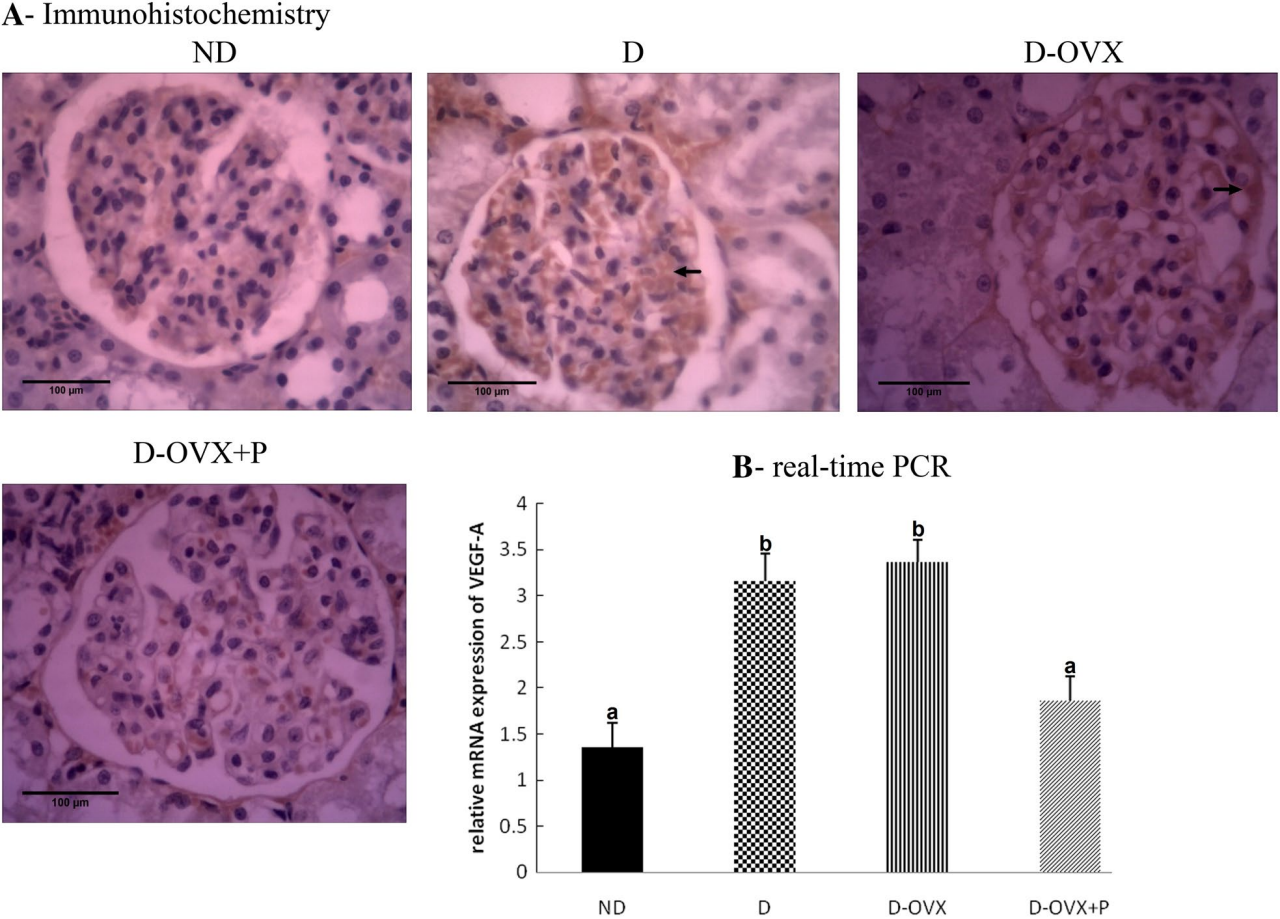
Immunohistochemistry and real-time PCR detect that progesterone replacement inhibits the VEGF-A expression in the diabetic kidney. **A** Immunohistochemical stain of the kidney sections (hematoxylin staining; magnification, ×400) show that the VEGF-A immunostaining (brown staining) in the glomeruli was much stronger in the D and the D-OVX groups compared with the ND group (*arrows*). Progesterone replacement inhibited the increase in the VEGF-A immunostaining in D + OVX + P group. **B** VEGF-A mRNA expression by real-time PCR. Data represent the mean ± SEM. Means with *different superscript letters* are significantly different from one another (*P* < 0.05)

Previous studies demonstrated a significant increase in VEGF-A mRNA expressions, an important growth factor for vascular development and angiogenesis, in the kidney of STZ-diabetic rats [22]. Therefore, we examined whether progesterone could influence the of kidneys expression level of VEGF-A in the diabetic rats. Figure 8A, B, shows that the mRNA expression and the protein levels of VEGF-A were significantly increased in the D and the D-OVX rats compared with the ND rats. The treatment with progesterone resulted in a marked reduction of the VEGF-A levels in the D-OVX + P rats as compared with the D and the D-OVX rats.

## Disscusion

Increasing evidence suggests that sex hormones (estradiol and dihydrotestosterone) are important regulators of the renal function and may be novel targets for the treatment and prevention of the diabetic renal disease in females and males, respectively [8, 23]. So far, the role that progesterone, the other female sex hormone, plays during the DN progression is still unclear. Therefore, the present study was intended to provide an insight into the specific effects of progesterone in diabetic renal complications. We demonstrate that in the STZ-induced diabetic ovariectomized rat, the replacement with progesterone for 10 weeks is renoprotective as evident from the restoration of various parameters as compared with the D and the D-OVX groups. In the present study, the administration of progesterone to the D-OVX rats significantly reduced UACR, glomerulosclerosis, the expression of profibrotic factors (TGF-β and fibronectin) and the expression of angiogenesis-associated factor (VEGF). Moreover, the progesterone replacement retains the expressions of podocyte markers (nephrin and podocin) and decreased ATR1 expression in the diabetic kidney.

The present work revealed that the treatment with progesterone could reduce the early manifestations of the DN as shown by a significant reduction of UACR, a key measure of the renal function [16], at 10 weeks after diabetes in treated diabetic animals compared with controls. A similar effect of progesterone on proteinuria has been observed in the ischemia–reperfusion–induced acute kidney injury [12]. The renal podocyte foot processes and associated slit diaphragms ensure the integrity of the renal basement membrane that forms the barrier to urinary protein loss [24]. To clarify the mechanisms of the beneficial effect of progesterone on proteinuria, we tested whether the progesterone replacement prevents downregulation of proteins required for slit diaphragm integrity, podocin and nephrin. After 10 weeks of diabetes, the development of albuminuria in the diabetic rats was associated with decreases in podocyte abundance and distribution which was manifested by reduced mRNA and protein expression of nephrin and podocin in diabetic animals. Our data indicated that treatment with progesterone restored mRNA expression and staining intensity of nephrin and podocin, suggesting a protective effect on the structure of podocytes. Hence, our data suggest that the effects of progesterone on attenuating proteinuria are possibly mediated by preventing the damage of podocytes in the diabetic conditions.

The expansion of the mesangial matrix and thickening of the glomerular basement membrane are the typical early presentation of DN [24]; and the current study confirms these findings. Both the glomerulosclerosis and tubulointerstitial fibrosis are characterized by the accumulation of ECM proteins as a result of the increased synthesis and/or the reduced degradative rates of ECM proteins [25]. In the diabetic kidneys, the overexpression of TGF-β is believed to be the most potent stimulator of ECM proteins accumulation [26]. Our results show that the renal TGF-β1 expression levels were higher in STZ-diabetic rats relative to the normal rats and the progesterone treatment attenuated this increase (Fig. 2). In addition, we have demonstrated that fibronectin, an important constituent of ECM proteins, was induced in the diabetic rats and the upregulation of tissue fibronectin in the diabetic kidney is effectively reversed after the progesterone treatment. Moreover, we observed that the progesterone treatment effectively increased the MMP-2 protein but not the mRNA levels in the diabetic kidney, indicating a posttranscriptional regulation of MMP-2 by the progesterone replacement. In this context, it has been shown that various exogenous and endogenous factors may regulate MMPs at the post-transcriptional level by stabilizing and/or destabilizing mRNA transcripts [27]. Taken together, the decrease in the expression of TGF-β1 and fibronectin in the diabetic kidney by the progesterone treatment, as well as the increase in the MMP-2 protein level clearly suggests that progesterone protects the development of renal fibrosis and glomerular mesangial matrix accumulation associated with the DN, at least in part, by inhibiting the ECM proteins production and enhancing the ECM proteins degradation.

Numerous studies revealed that in the hibition of intrarenal renin-angiotensin system (**RAS**) by using the angiotensin-converting enzyme inhibitors and/or the ATR1 blockers can attenuate progressive glomerulosclerosis in renal disease models and can slow the progression of renal disease in patients with type 2 diabetes [28]. Independent of its hemodynamic action, the exposure of mesangial cells to Ang II results in stimulation of TGF-β expression and secretion which leads to the increased synthesis and the decreased degradation of ECM proteins [28]. These data support the notion that Ang II can worsen the DN. The effects of Ang II are mediated by two plasma membrane Ang II receptors (ATR1 and ATR2). Most of the known effects of Ang II in adult tissues are attributable to the ATR1 [29]. Therefore, in this study we investigate the role of progesterone in the ATR1 expression in the kidney under diabetic conditions. Consistent with previous studies [21, 30], the present study found that ATR1 mRNA and protein expression levels were upregulated in the diabetic kidney, suggesting a potential role for ATR1 in the development and progression of renal injury secondary to diabetes mellitus. Because the progesterone replacement significantly reversed the upregulation of ATR1 in the diabetic kidney, we postulated that the renal protective actions of progesterone may be related, at least in part, to the decrease in the susceptibility of diabetic kidney to the available circulating and locally produced Ang II, and hence blocks the local activation of RAS. Certainly, this hypothesis needs to be tested by further studies.

There is a growing body of studies showing that angiogenesis may play an important role in the pathogenesis of the DN. Angiogenesis is associated with glomerular hypertrophy and urinary albumin excretion in the DN [31]. Additionally, previous studies have indicated that blocking angiogenesis attenuated the glomerular basement membrane thickening, the mesangial expansion, and the TGF-β expression in diabetic animals [31, 32]. The angiogenic growth factor VEGF-A is induced by high glucose levels in the early phases of diabetes, leading to the development of early features of the DN [31, 33]. In the present study, the level of VEGF-A was markedly increased in the kidney of the diabetic rats, which is consistent with previous studies [2, 33]. We demonstrated also for the first time that progesterone could attenuate the DN via inhibiting the angiogenesis-associated factor VEGF-A. These findings suggest that blocking the angiogenesis induced by VEGF-A may play an important role in the renal benefit of progesterone in the diabetic rats.

Progression of diabetic renal disease in STZ-diabetic male rats has been reported by some authors to be correlated with the lower ratio of testosterone to oestradiol or progesterone rather than with absolute levels of sex hormones [34]. In the present work we measured the concentrations of progesterone and estrogen without testosterone. However, given the fact that experimental diabetes leads to increased plasma testosterone levels in the adult female rat [3, 35], mainly from adrenal origin [35], we speculated that increase rather than decrease in the ratio of testosterone to oestradiol or progesterone is correlated with the severity of the diabetic renal disease in the female STZ-induced diabetic rat. Further studies are warranted to examine the effects of testosterone in the pathophysiology of the diabetic renal disease in a female animal model of diabetes.

Finally, many hormonal abnormalities and sexual dysfunctions which occur in women with diabetes may be treated with progesterone alone or estrogen-progesterone combinations [36, 37]. Although our report is an experimental study of DN in the rat, we provide a proof of concept evidence that progesterone may be effective in controlling both the sexual dysfunction and the DN in the diabetic women.

In conclusion, our study is the first to demonstrate that the progesterone replacement can ameliorate renal damage in experimental models of the DN through improving the renal function; inhibition of renal fibrosis and angiogenesis; along with amelioration of podocyte injury. We demonstrated also that progesterone could decrease the Ang II effect in the DN through decrease the ATR1 expression. These findings suggest that progesterone replacement may have a therapeutic potential in the DN.

## Additional files

10.1186/s13098-015-0097-1equences of primers used for quantitative real time RT-PCR. 
10.1186/s13098-015-0097-1 Body weight changes in the control and the diabetic rats. After 10 weeks of diabetes, all diabetic groups showed a significant decrease in body weight as compared with the ND group. Data represent the mean ± SEM.*P < 0.05 compared to the ND group.
