# Supplementary material for: Progesterone ameliorates diabetic nephropathy in streptozotocin-induced diabetic Rats
Source: Diabetol Metab Syndr. 2015 Nov 14;7:97. doi: 10.1186/s13098-015-0097-1 (PMC4650109; doi:10.1186/s13098-015-0097-1)
Supplement: Supplementary file 1 — 10.1186/s13098-015-0097-1equences of primers used for quantitative real time RT-PCR. [file 13098_2015_97_MOESM1_ESM.doc]

| **Table 1.** Sequences of primers used for quantitative real time RT-PCR | | | |
| --- | --- | --- | --- |
| Gene^1^ | GenBank accession | Forward (5–3) | Reverse (5–3) |
| β-Actin | NM_031144 | CCTAGACTTCGAGCAAGAGA | TCCATACCCAGGAAGGAAG |
| TGF-β | NM_021578 | CGTACACAGCAGTTCTTCTCT | ATGACATGAACCGACCCTTC |
| fibronectin | X15906.1 | TCACAGGGTACAGGATTGT | TCTCCTCCACAGCATAGATAG |
| MMP-2 | NM_031054 | GAACACAGCCTTCTCTTCCT | GTTTATTTGGCGGACAGTGAC |
| podocin | NM_130828.2 | ATTCCGACTGGGACATCT | GTTACCACCTCATGGAAAGG |
| nephrin | AF172255.1 | CATTATGCTCCCACCATCC | TCAGATCCTCCTCTTCTTCTC |
| ATR1 | NM_030985.4 | CACTATTCGAAATCCACTTGACC | CTCTCAGCTCTGCCACATTC |
| VEGF-A | NM_031836 | TTTCCCTTTCCTCGAACTGAT | ACGTCACTATGCAGATCATGC |
| ^1^TGF-β: transforming growth factor- β; MMP-2: matrix metalloproteinases-2; ATR1: angiotensin II type 1 receptor; VEGF-A: vascular endothelial growth factor-A. | | | |
