# Supplementary figures and images for: Progesterone ameliorates diabetic nephropathy in streptozotocin-induced diabetic Rats
Source: Diabetol Metab Syndr. 2015 Nov 14;7:97. doi: 10.1186/s13098-015-0097-1 (PMC4650109; doi:10.1186/s13098-015-0097-1)

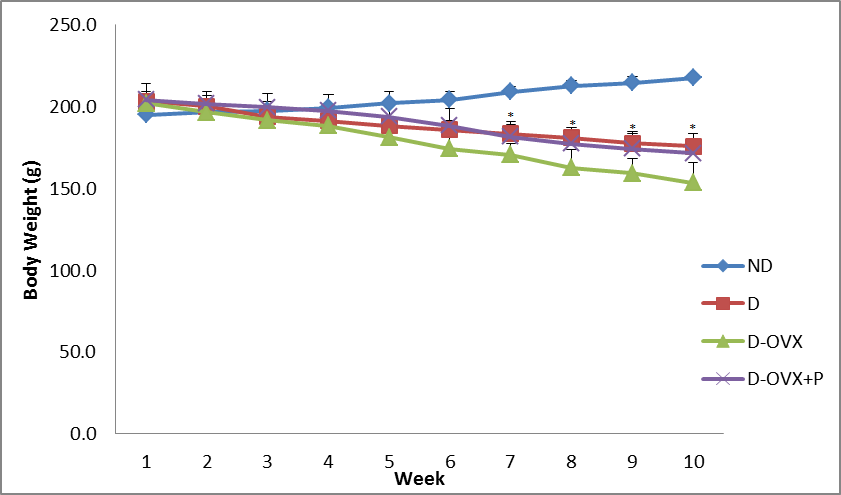

Supplement: Supplementary file 2 — 10.1186/s13098-015-0097-1 Body weight changes in the control and the diabetic rats. After 10 weeks of diabetes, all diabetic groups showed a significant decrease in body weight as compared with the ND group. Data represent the mean ± SEM.*P < 0.05 compared to the ND group. [file 13098_2015_97_MOESM2_ESM.docx]
